# Frequency of Vitamin D Deficiency in Patients of Asthma

**DOI:** 10.7759/cureus.14828

**Published:** 2021-05-03

**Authors:** Souhaib Alvi, Jabbar Ghufran Syed, Baakh Nusrat, Syed Kumail Abbas Razvi, Zunaira Z Shah, Yusra Shafaat khan, Muhammad Danish Khan, Muhammad Ali Khan

**Affiliations:** 1 Medicine, Abbassi Shaheed Hospital, Karachi, PAK; 2 General Surgery, Jinnah Postgraduate Medical Centre, Karachi, PAK; 3 Medicine, Ziauddin University, Karachi, PAK; 4 Orthopedics, The Indus Hospital, Karachi, PAK; 5 Gastroenterology, Jinnah Postgraduate Medical Centre, Karachi, PAK

**Keywords:** vitamin-d deficiency, asthma, frequency, karachi, pakistan

## Abstract

Introduction

Asthma is a clinical syndrome characterized by reversible and recurrent airway obstruction leading to the symptoms of wheezing, cough, shortness of breath, fatigue, and respiratory distress. It is one of the most common lung pathologies worldwide. Its incidence is on the rise in Pakistan, which may be due to overt environmental pollution or improving screening protocols. Irrespective more and more patients are now being diagnosed as cases of asthma and this has led to renewed interest in research for the subject locally.

Vitamin D plays a key component of the immune system and its deficiency has been associated with diseases, such as inflammatory bowel disease, celiac disease, rheumatoid arthritis, depression, sepsis, and coronavirus disease 2019 (COVID-19) pneumonia. The role of vitamin D in exacerbation, prevention, causation, and treatment of asthma is still up for debate. However, as more data emerges, it is becoming evident that vitamin D in one way or another is linked to outcomes in asthma. Especially the deficiency of vitamin D in asthma and its supplementation has garnered great attention in scientific trials. Our research is just one small step in this direction. This study aims to evaluate the frequency of vitamin D deficiency in patients with asthma.

Methods

This was a retrospective cross-sectional cohort conducted at the Department of Medicine of Abbasi Shaheed Hospital Karachi (ASHK) during March 2019 to August 2019. Patients of either gender aged ≥18 years that were diagnosed with cases of asthma were eligible to be included in the study. Asthma was defined in accordance with the latest guidelines issued by the Pakistan Chest Society. A vitamin D level of <20 ng/dl was considered deficient. Patient confidentiality was made certain.

Results

A total of 97 (62.98%) of the 154 patients included in the study had <20 ng/ml of vitamin D level. The mean vitamin D level recorded in this study was 15.34±4.21 ng/dl. The male-to-female ratio was 1:5. The mean age of our cohort was 42.78±4.56 years. The mean duration of disease, i.e., asthma was 6.7±3.68 years. Both the age of the patients and duration of the disease were found to be statistically significant with respect to vitamin D levels in asthmatics.

Conclusions

The frequency of vitamin D deficiency is very high in patients with asthma. These suboptimal levels are significantly influenced by the age of the patient and the duration of the disease.

## Introduction

Asthma is one of the most prevalent pulmonary pathologies around the world [1]. Its incidence is on the rise in Pakistan [2]; this could be due to the improved practices of screening and diagnosis or simply a result of overt environmental pollution [3]. Over the last quarter of a century, advancements have been made in treatment and intervention that have improved the quality of life for asthmatics substantially [4]. However, the health care cost for management of asthma is still very high; a major share of the morbidity and mortality associated with asthma is due to improper drug administration, unavailability of drugs, noncompliance by the patients, drug side-effects (specifically with steroids), misconceptions about the disease, cultural beliefs, device-related mishaps, and underlying undiagnosed comorbid [5]. Predisposing factors including smoking, obesity, poverty, residence in a high pollution zone, and malnutrition all contribute to poor outcomes as well [6].

Vitamin D is a key component of the immune system and its deficiency has been reported in inflammatory bowel disease, rheumatoid arthritis, celiac disease, clinical depression, encephalomyelitis, sepsis, and most recently in patients with COVID-19 pneumonia [7-10]. The prevalence of vitamin D deficiency is high in Pakistan and does not demonstrate significant seasonal disparity for gender, comorbid, or age; it is a phenomenon consistently observed by health care workers throughout the year [11,12]. As such it remains difficult to correlate vitamin D levels with any disease or its progress as so many of the population harbor suboptimal levels usually without any sign or symptom [12]. None the less vitamin D deficiency is a marker of malnutrition and therefore can be a contributor to any disease process.

Pfeffer and Hawrylowicz previously described the benefits of optimization of vitamin D levels in the asthma patient leading to improved outcomes [13]. Other reports have indicated the role of vitamin D (or its deficiency) in the development, exacerbation, prevention, causation, and treatment of asthma; the exact mechanism by which vitamin D influences these processes remains unascertained and the data so far is conflicting [14-16]. Ergo, it is not possible to establish scientific conclusions with respect to this matter and our knowledge of the subject is still evolving as of writing.

As mentioned before, the prevalence of vitamin D deficiency is very high in Pakistan [11]. At these rates, it is not uncommon to unearth subpar vitamin D levels even in asymptomatic and apparently “healthy” individuals [17]. The incidence of vitamin D deficiency is still higher among patients with a history of atopy and allergies in the Pakistani population; the incidence of vitamin D deficiency was reported to be as high as 58% in these patients very recently [18]. Atopy and allergic rhinitis are closely linked with asthma through complex pathophysiological pathways; therefore finding abnormal vitamin D levels in asthmatics wouldn’t be unsurprising [19]. Unfortunately, local data are scarce in this matter and we hope to mitigate this by evaluating vitamin D levels in patients with asthma.

## Materials and methods

This was a retrospective cross-sectional cohort conducted during March 2019 to August 2019. It was held at the Department of Medicine of Abbasi Shaheed Hospital Karachi (ASHK). All data were collected from the records section of the Department of Medicine. Patient confidentiality was made certain at all times. Patients aged ≥18 years of either gender that were diagnosed with cases of asthma were eligible to be included in the study. Asthma was defined according to the latest guidelines issued by the Pakistan Chest Society (PCS) at the time this research was being carried out; these guidelines have since been updated [20].

Asthma was defined by the history of typical symptoms such as wheezing, shortness of breath, cough, or chest tightness with variation to time and place for a minimum of two months along with the demonstration of airflow limitation on pulmonary function tests, i.e., forced expiratory volume (FEV1) of less than 80% of that predicted, and a forced expiratory volume/forced vital capacity (FEV1/FVC) ratio of <0.7. Additional investigations such as radiological imaging, allergy testing, nitric oxide test, sputum eosinophils, methacholine challenge, and provocative testing for exercise were also accepted as additional proof of asthma. Neither the severity levels of asthma nor the types of asthma i.e. allergic versus nonallergic were evaluated for this study. Vitamin D levels were analyzed using the COBAS (Roche Diagnostics, Risch-Rotkreuz, Switzerland) machine. A level of <20 ng/dl was taken as deficient. BMI was calculated by dividing the weight of the patient in kilogram(s) by height in meter(s) squared.

Patients with chronic decompensated liver disease, renal impairment (creatinine >1.5 mg/dl), those taking vitamin D or calcium supplements, taking medications for tuberculosis, patients on chemotherapy, immunocompromised patients, patients who had undergone parathyroidectomy, and patients with underlying parathyroid and/or thyroid disorders were excluded from the study.

Data was entered on Statistical Package for the Social Sciences (SPSS) for Windows, Version 21.0. Chicago, SPSS Inc. The consecutive and nonprobability sampling technique was used for this study. Mean and standard deviation were calculated for quantitative variables of age, vitamin D levels, duration of asthma, and BMI. Frequency and percentages were computed for qualitative variables of gender, age groups (<40 or ≥40 years), duration of asthma (<5 or ≥5 years), and BMI (<25 or ≥25 kg/m^2^). A chi-square test was used. A p-value of ≤0.05 was considered significant.

## Results

Of the 154 patients enrolled in the study, the majority were female; the male-to-female ratio was approximately 1:5. The mean age of our cohort was 42.78±4.56 years. The mean duration of disease, i.e., asthma was 6.7±3.68 years, more than two-thirds of the patient had the disease for more than five years. The mean vitamin D level recorded in this study was 15.34±4.21 ng/dl. Ninety-seven (62.98%) patients had a vitamin D level of <20 ng/ml. The majority of the patients had a BMI of ≥25 kg/m^2^. General characteristics, duration of asthma, vitamin D levels, and BMI of the patients included in the study are summarized in Table 1. 

**Table 1:** General and biochemical characteristics of the patients included in the study.

	N=154
Age (mean)	42.78±4.56 years
Age groups	
<40 years	85(55.2%)
≥40 years	69(44.8%)
Gender	
Female	125 (81.2%)
Male	29 (18.8%)
Duration of asthma (mean)	6.7±3.68 years
Duration of asthma ≥5 years	105 (68.2%)
Duration of asthma <5 years	49 (31.8%)
Body mass index (mean)	25.76±3.61 kg/m^2^
Body mass index <25 (kg/m^2^)	42 (27.27%)
Body mass index ≥25 (kg/m^2^)	112 (72.72%)
Vitamin D levels ng/dl (mean)	15.34±4.21
Vitamin D level <20 ng/dl	97 (62.98%)
Vitamin D level ≥20 ng/dl	57 (37.01%)

Eight-seven (56.49%) females, more than half of the cohort had vitamin D deficiency, by contrast only 10 (6.49%) male patients had vitamin D deficiency. However, post-stratification gender was not found to be a statistically significant factor with respect to vitamin D deficiency in patients with asthma; similar trends were seen with BMI. On the other hand, older patients and those with a longer duration of disease had statistically significant vitamin D deficiency. Subgroup analysis is shown in Table 2.

**Table 2:** Subgroup analysis of pertinent variables with respect to vitamin D deficiency.

Parameter	Vitamin D deficiency N (%)		p-Value
	Yes	No	
Gender			
Male	10 (6.49%)	19 (12.33%)	0.66
Female	87 (56.49%)	38 (24.67%)	
Duration of disease			
Asthma duration <5 years	16 (10.38%)	33 (21.42%)	0.005
Asthma duration ≥5 years	81 (52.59%)	24 (15.58%)	
Age group			
Age ≥40 years	59 (38.31%)	10 (6.49%)	0.001
Age <40 years	38 (24.67%)	47 (30.51%)	
Body mass index			
Body mass index ≥25 kg/m^2^	72 (46.75%)	40 (25.97%)	0.17
Body mass index <25 kg/m^2^	25 (16.23%)	17 (11.03%)	

## Discussion

The incidence of allergic asthma is highest during childhood and adolescence while the incidence of nonallergic asthma increases with age and has predominance for the female gender; most cases of nonallergic asthma are diagnosed between the ages of 38-53 years with a peak incidence in the fourth decade of life [21]. The prevalence of nonallergic asthma is much higher in the Pakistani population due to signal transducer and activator of transcription 6 (STAT6) polymorphisms [22]. This would explain the relatively high mean age of the patients recorded in the study. Approximately 55% of the patients were 40 years or younger, most in this group were in their mid-thirties, contributing to a mean age of 42.78±4.56 years. Only a handful of the patients were diagnosed with asthma in their childhood or adolescence. Nonallergic asthma has a higher prevalence among women, but the inordinately high number of female patients included in our cohort had more to do with vitamin D deficiency rather than the type or nature of asthma as we discuss below.

Since nonallergic asthma is mostly diagnosed in the fourth decade of life, the duration of disease/illness corresponded accordingly to the age of our patients. The median age and duration of illness in our study were 45 years and five years, respectively. Nearly 70% of the patients had asthma for a time period of more than five years and with a few exceptions just about all patients had asthma for a minimum of three years. However, It is critical to remember that the duration of disease was recorded since asthma was diagnosed on pulmonary function tests and/or on imaging; there is a disconnect between the onset of the symptoms of asthma and its diagnosis in Pakistan due to several reasons, and it would not be surprising if the real-life duration of the disease in most patients were much longer.

Asians are at a greater risk of adverse cardiovascular events and type 2 diabetes compared to their European counterparts for the same levels of BMI, but no clear cut-off BMI has been decided upon by the WHO with respect to this matter [23]. The risk of adverse events varies in the Asian population from a BMI of 22 kg/m^2^ to 25 kg/m^2^, however, patients with a BMI of >25 kg/m^2^ have consistently been observed to have a high risk of adverse events irrespective of geographical location (within the Asian continent) [23]. As of now, the cut-off for obesity remains at >25 kg/m^2^ for all ethnicities and it was concerning to record such a high frequency of obesity in our study. The mean BMI of 25.76±3.61 kg/m^2^ recorded in our cohort is congruous with antecedent data [21,24]. Lampalo et al. not only recorded higher BMI values for asthmatics compared to controls but also identified BMI as an independent risk factor for nonallergic asthma in females [24], similar results were also reported by Wang et al. [25]. However, both Lampalo et al. and Wang et al. reported much higher levels of obesity (mostly grade II or >30 kg/m^2^) than ours. Perhaps this was the one reason that in our study BMI did not reach statistical significance for either the incidence of asthma or reduced vitamin D levels in asthmatics.

International and local authors have described exceedingly high rates of vitamin D deficiency among adult asthmatic patients [26,27]. Kamran et al. reported vitamin D deficiency in 67.66% of the female patients and just over half of the male patients in his cohort conducted at Jinnah Postgraduate Medical Centre (JPMC), Karachi [27]. These results are quite similar to ours. However, such high rates of vitamin D deficiency are not unique to asthma in Pakistan. Studies have found similar or higher rates of vitamin D deficiency in healthy controls; Mehboobali et al. reported vitamin D deficiency rates of 58.4% in random healthy individuals most of whom did not have any comorbid at all [28]. Jadoon et al. reported vitamin D deficiency in 81.7% and 18.3% of female and male subjects respectively [17]. Unlike in our analysis, the studies discussed above demonstrated statistically significant vitamin D deficiency in females compared to males.

The overall rate of vitamin D deficiency for females in our study was 56.49%, this is lower than what was reported by both Kamran et al. and Mehboobali et al., but the relative rate of vitamin D deficiency for females was approximately 70% in our study, which is higher than reported in either study [27,28]. While the relative rates of vitamin D deficiency were found to be statistically significantly affected by gender, the overall rates once stratified did not have such statistical significance. Therefore it stands to reason that while gender alone plays an important role in vitamin D deficiency, it does not have always have the same importance in asthmatics, where its impact is affected by other variables. These studies also explain the disproportionate number of females encountered in our cohort.

Both the age of the patients and duration of the disease were found to be extremely important with respect to vitamin D levels in asthmatics. The age and duration of the disease represent a single continuum of the disease. Both are correlated and proportional. The longer the duration of the disease the more aged the patient will be. This is especially true for nonallergic asthma, as most cases are diagnosed during the third, fourth, or fifth decades of life [21]. Factors previously investigated for markedly reduced levels of vitamin D in asthmatics include use of steroid, altered metabolism of vitamin D, depression, oxidative stress, lack of physical activity, reduced exposure to sunlight, and malnutrition [14,29,30]. Given sufficient time any of these may lead to pathological levels of vitamin D and in the author’s opinion until definite data comes out in the future, this remains the best explanation as to why the increased duration of disease in asthma can cause significantly reduced levels of vitamin D. But, as of now, no definitive link has been found between vitamin D deficiency and any of the aforementioned factors in asthma. Both age and duration of disease are nonmodifiable variables. There is not much that can be done to ameliorate their impact. For the time being, vitamin D supplementation has provided us with the most promising results.

Data from recent trials and reviews suggest that vitamin D supplementation is safe and beneficial in patients with asthma [13,15]. Evidence is mounting in favor of vitamin D supplementation but complete knowledge of specific biological workings is still missing that may explain certain negative results. Future trials need to target the exact mechanisms by which vitamin D influences the various pathophysiological processes in asthma and how vitamin D supplementation can be used as a therapeutic tool to improve outcomes.

Limitations of the study

Complete nutritional assessment was not performed in any of the patients. The impact of medications being used by the patients such as steroids, inhalers, histamine receptor blockers, proton pump inhibitors, etc. was not analyzed at all. Smoking history was not assessed in the study. Comorbid were not recorded.

## Conclusions

The frequency of vitamin D deficiency is very high in patients with asthma. The reduced vitamin D levels are significantly affected by the duration of the disease and the age of the patient. Further research is required to ascertain if these greatly reduced levels are specific to asthma and its disease process or point to a greater underlying medical problem in the Pakistani population.
